# Template-assisted, Sol-gel Fabrication of Biocompatible, Hierarchically Porous Hydroxyapatite Scaffolds

**DOI:** 10.3390/ma12081274

**Published:** 2019-04-18

**Authors:** Xingyuan Zhang, Lirong Zhang, Yuanwei Li, Youlu Hua, Yangde Li, Weirong Li, Wei Li

**Affiliations:** 1 School of Mechanical Engineering, Liaoning Technical University, Fuxin 123000, China; zxyuan001@163.com (X.Z.); zhanglirong024@126.com (L.Z.); 2 Institute of Advanced Wear & Corrosion Resistant and Functional Materials, Jinan University, Guangzhou 510632, China; jnulyw@stu2016.jnu.edu.cn (Y.L.); liweijnu@126.com (W.L.); 3 Eontec Co., Ltd., Dongguan 523000, China; chiefdesigner@e-ande.com

**Keywords:** hydroxyapatite, biomaterials, scaffolds

## Abstract

Hierarchically porous hydroxyapatite (HHA) scaffolds were synthesized by template-assisted sol-gel chemistry. Polyurethane foam and a block copolymer were used as templates for inducing hierarchically porous structures. The HHA scaffolds exhibited open porous structures with large pores of 400–600 µm and nanoscale pores of ~75 nm. In comparison with conventional hydroxyapatite (CHA), HHA scaffolds exhibited significantly higher surface areas and increased protein adsorption for bovine serum albumin and vitronectin. Both the HHA and CHA scaffolds exhibited well in vitro biocompatibility. After 1 day, Saos-2 osteoblast-like cells bound equally well to both HHA and CHA scaffolds, but after 7 days in culture, cell proliferation was significantly greater on the HHA scaffolds (*p* < 0.01). High surface area and hierarchical porous structure contributed to the selective enhancement of osteoblast proliferation on the HHA scaffolds.

## 1. Introduction

Due to its chemical and biological similarity to the mineral phase of human bone, synthetic hydroxyapatite (HA) exhibits direct chemical bonding to hard tissue (osteointegration) and is bioactive. Consequently, HA has been widely used for a variety of biomedical applications, including mid-ear and bone implants, artificial vertebrae, and as orthopedic coatings [1,2,3,4]. Bioactive materials that are also resorbable have gained attention as candidate materials for tissue engineering scaffolds [5,6,7,8]. It is widely considered that the scaffolds used for this application should be highly porous, three-dimensional (3D) structures with interconnected large pores (>300 µm), in order to facilitate cell growth and the diffusion of nutrients and metabolic waste. Surface chemistry appropriate for the attachment, proliferation and differentiation of cells, and mechanical properties to match the surrounding tissues are also required. One popular technique to create open porosity in materials is to incorporate pore creating additives (porogens). These porogens can be crystals or particles of either volatile or soluble substances, such as paraffin [9], NaHCO_3_ [10,11], gelatin [12,13], or polymethylmethacrylate (PMMA) [14]. Open porosity may also be created by replication [6], 3D printing techniques [8,15], a direct foaming technique, or hydrothermal conversion of cuttlebone. These methods inevitably lead to large crystal size and low surface area for various reasons. In contrast, sol-gel synthesis of HA offers fabrication advantages, which include high chemical purity, homogeneous composition, and low synthesis temperature. Sol-gel chemistry has been used to prepare nanocrystalline HA powders [3], surface coat HA thin films onto metal substrates and for the self-forming synthesis of porous HA materials [16,17]. However, HHA is non-resorbable, unless it is nanometric [18].

Mesoporous materials, which have pores in the range of 2 to 50 nm, have been proposed for use in drug delivery and bone tissue engineering applications. Highly ordered 2D hexagonal or 3D cubic mesoporous bioactive glasses with superior in vitro bioactivity have been reported [19,20,21]. The associated high surface area and pore volume of these materials are favorable for protein adsorption and also promote cell adhesion and proliferation. By introducing pores in the nanometer range into scaffolds, the degradation of scaffolds may be controlled to match the rate of new tissue growth.

In the present work, a highly porous polyurethane (PU) foam, with interconnected, open large pores, and a tri-block copolymer were used to create interconnected porosity on the microscale and nanoscale, respectively, in the final HA scaffold. The synthesized hierarchically porous hydroxyapatite (HHA) scaffolds exhibited superior physical and biological properties compared to conventional hydroxyapatite (CHA). Hence, HHA scaffolds were found to be suitable materials for bone tissue engineering. Additionally, this combined templating and sol-gel chemistry approach could potentially be adopted to synthesize other porous biomaterials, depending on the templates used. Templating is a flexible and reproducible process for the formation of porous scaffolds with controlled porosity and pore size.

## 2. Materials and Methods

### 2.1. Materials

All the chemicals were used as received from the manufacturer. For the preparation of the HA materials, calcium nitrate tetrahydrate (Ca(NO_3_)·4H_2_O, AR), triethyl phosphate (P(OC_2_H_5_)_3_, AR), diammonium hydrogen phosphate ((NH_4_)_2_HPO_4_, AR), Pluronic F127, polyvinyl alcohol (PVA), and 10× concentrated phosphate buffered saline (10× PBS) all came from Sigma-Aldrich, while anhydrous ethanol was from Merck (Darmstadt, Germany). Protein adsorption and cell culture studies required bovine serum albumin (BSA), vitronectin, BCA^TM^ Protein Assay Kit, hexamethyldisilane (HMDS), and sodium dodecyl sulphate (SDS, AR) all from Sigma-Aldrich; Minimum Essential Media (MEM), Glutamax, Non-Essential Amino Acids (NEAA), Antibiotic-Antimycotic solution (Anti-Anti), and TrypLE Express were purchased from Invitrogen; MTS reagent from Promega, and Fetal Bovine Serum (FBS) from SAFC Biosciences (Hampshire, England). The water used in all experiments was passed through a Millipore (Boston, MA, USA) Milli Q ultrapure water purification system and had a resistivity higher than 18.2 MΩcm.

#### Hydroxyapatite Scaffold Fabrication

HHA scaffolds: To prepare the HA sol, 7.47 g P(OC_2_H_5_)_3_ was hydrolyzed with 6.48 g water (molar ratio 1:8) by vigorous stirring for 24 h. 17.74 g Ca(NO_3_)·4H_2_O and 1 g Pluonic F127 was dissolved in 25 mL ethanol with stirring for 24 h. This solution was added dropwise to the hydrolyzed P(OC_2_H_5_)_3_. The mixture was aged in a sealed bottle at 60 °C for 24 h. The solvent was allowed to evaporate in a fume cupboard at room temperature for 48 h, resulting in a viscous gel. PU foam templates (density 25 ppi) were cut into blocks of approximately 8 × 8 × 12 mm^3^ or discs of Ø 10 mm × 5 mm. The PU foam pieces were completely immersed in the viscous gel and compressed in order to remove air and infiltrate the sol into the pores. After drying at room temperature for 1 day, the procedure was repeated twice more. The coated scaffolds were then dried in air at room temperature for 7 days and then at 60 °C for 24 h. The samples were heated under flowing air at a rate of 2 °C min^−1^ to 400 °C, held for 1 h, then at 5 °C min^−1^ to 720 °C, and held for 5 h.

CHA scaffolds: First HA nanoparticles were synthesized by a chemical precipitation method [22]. Briefly, aqueous 0.1 M Ca(NO_3_)_2_ (100 mL) was dropped into an aqueous 0.06 M (NH_4_)_2_HPO4 solution (100 mL) under stirring for 12 h and maintained at pH >10. The resulting powders were filtered and washed three times in water and once in ethanol, then dried and calcined under flowing air at 800 °C for 2 h.

CHA scaffolds were fabricated using a replication method [6], whereby 30 g HA nanoparticles were suspended in 100 mL aqueous solution containing 1 wt.% PVA and stirred to obtain a well-dispersed slurry. The prepared PU foam was immersed in a glass beaker containing the HA slurry and compressed with a glass stick to remove air and infiltrate the HA slurry into the pores of the foam. After drying at room temperature for one day, the procedure was repeated twice more. The impregnated sponge was dried at 60 °C for 1 day, and then sintered under flowing air at 1100 °C for 3 h, using a heating rate of 2 °C min^−1^.

### 2.2. Materials Characterization

The porous structure of the scaffolds was characterized by optical microscopy (Leica APO) and scanning electron microscopy (FE-SEM, Philips XL30, Holland, Amsterdam). The phase composition of the materials was determined by powder X-ray diffraction (XRD, Bruker D8, Karlsruhe, Germany). The general porosity, ε, of the materials was calculated by the formula [11]:

(1) $$\varepsilon =\left(1-\frac{\rho }{\rho_{s}}\right)*100,$$

where ρ and $\rho_{s}$ are the density of the porous HA and the theoretical density of hydroxyapatite, 3.14 g cm^−3^ [23], respectively. The density of the porous HA was determined from its mass and dimensional measurements. SEM images combined with quantitative image analysis through Image-Pro Plus (Media Cybernetics, Inc., Silver Spring, MD, City, State, Country) software were used to determine the pore size distribution. Nitrogen sorption isotherms were carried out on a Micromeritics Tristar 3020 analyzer (City, State, Country) at 77 K. Prior to the measurement, the samples were degassed at 150 °C on a vacuum line for 18 h. The standard multipoint Brunauer-Emmett-Teller (BET) method was utilized to calculate the specific surface area using the adsorption data in the P/P_0_ range from 0.05 to 0.20. The pore size distributions of the materials were derived from the adsorption branch of the isotherms on the basis of the Barrett-Joyner-Halenda (BJH) model. The compressive strength of the scaffolds (8 × 8 × 12 mm^3^) was measured using an Instron 5566 mechanical tester at a cross-head speed of 0.5 mm min^−1^ with a 1 kN load cell. To evaluate the degradation process of the HHA scaffolds, the scaffolds were immersed in PBS for 1, 3, 7 and 14 days. At the given times, the scaffolds were taken out from the PBS and dried at 100 °C for 1 day, and the final mass of each sample was carefully measured. The compressive strength of the scaffolds was tested and porosity was calculated using the same method described above. The compression tests were done on five identical scaffolds and the results were expressed as a mean ± standard deviation.

### 2.3. Protein Adsorption

Protein adsorption experiments were carried out in 15 mL capacity polypropylene centrifuge tubes. The mixtures of scaffold material (crushed, 20 mg) and 6 mL protein solutions (BSA or vitronectin, 0.2 mg mL^−1^) were incubated at room temperature for 4 h with continual agitation to suspend the particles, and then the supernatants were removed after 5 min centrifugation at 10,000 rpm. The particles were washed three times with 10 mL water to remove loosely bound proteins by re-suspension and centrifuging for 3 min. After washing, 10 mL of 2% (w/v) SDS solution was added to the tubes to desorb the proteins attached to the surface with rapid shaking for 1 min. The suspensions were centrifuged for 5 min (10,000 rpm) and the supernatants containing the desorbed protein were collected for protein quantitative analysis by the BCA method [24]. The amount of desorbed protein in solution was determined by UV-visible spectroscopy at 562 nm. All experiments were carried out in triplicate.

### 2.4. Cell Culture

The scaffolds (both CHA and HHA) were washed in water and autoclaved prior to cell culture experiments, which were performed in 24 well non-treated tissue culture plates. Saos-2 osteoblast-like cells (10,000 per well) were cultured in MEM with Earl’s salts and Glutamax supplemented with 10% FBS, 1% NEAA and 1% antibiotic solution and maintained at 37 °C in a humidified atmosphere of 5% CO_2_ and 95% air. The media was changed every 2 days. For cell proliferation studies using MTS assay, the Live/Dead viability and SEM observations, the cells were trypsinised, centrifuged and counted. A 50 µL cell suspension containing 10,000 cells was dropped onto the top surface of each scaffold (Ø 10 mm × 5 mm) and cells were allowed to adhere on the scaffolds for 3 h in the incubator. Further, 1 mL of complete media was added to each well. Each scaffold was evaluated in triplicate and control tissue culture plastic plates were run in parallel for both MTS and Live/Dead^®^ assay.

The MTS assay for determining cell proliferation required two identical plates to be set up for harvesting after 1 and 7 days. Standard curves of known cell numbers were also established in conjunction with each time point. Briefly, the media was removed and the MTS solution was added to each well and incubated for 4 h at 37 °C. Absorbance of this solution was recorded at 490 nm using a plate reader (Bio-Tek, Power Wave XS, Winooski, VT, USA) with KC4 software. Triplicate colorimetric analysis and comparison to a standard curve of known viable cell numbers were used to calculate viable cell numbers for each condition. Significant differences in the cell number were analyzed using one-way ANOVA (*p* < 0.01).

Cell viability was assessed using the Live/Dead^®^ Kit (Mo-lecular Probes) which stains live cells with Calcein AM and dead cells with ethidium homodimer-1. Cell-scaffold constructs were gently washed twice with warm PBS and incubated with Live/Dead^®^ stain solution for 30 min at 37 °C. The constructs were analyzed using fluorescence microscopy (Nikon Eclipse TE2000-U, City, State, Country) and NIS-Elements image software.

For SEM, the cell-seeded discs after culture were fixed in 3.9% glutaraldehyde for 12 h at room temperature. Then the cells were dehydrated through sequential washings in 60, 70, 80, 90, 95 and 100% ethanol solutions for 10 min at each step. Next, the samples were chemically dried using HMDS and coated in gold for SEM observations.

## 3. Results and discussion

### 3.1. Open Porous Structure

**Figure 1:**
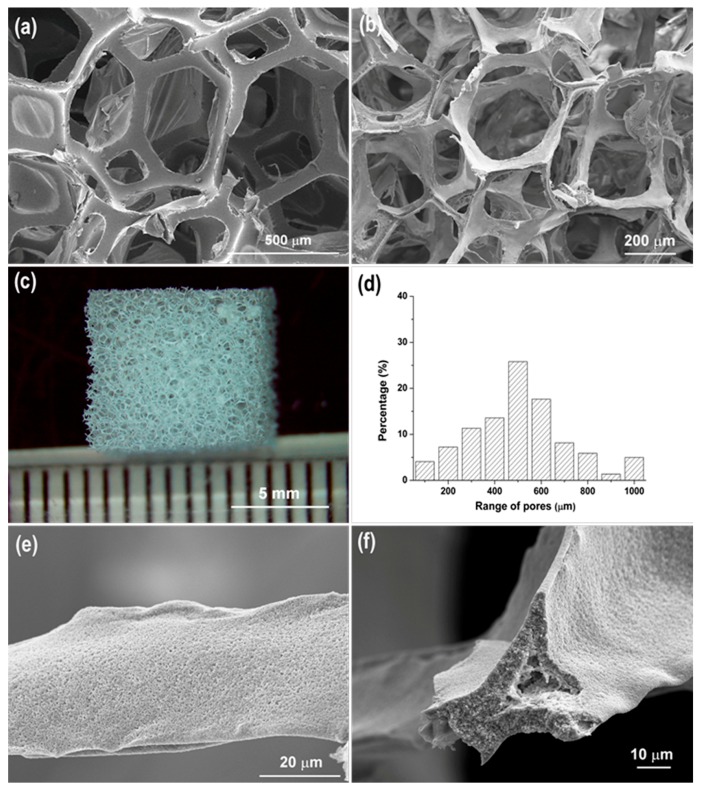
(**a**) SEM image of the PU foam; (**b**) SEM image of the HHA scaffold; (**c**) optical images of the HHA scaffold; (**d**) Pore size distribution of HHA scaffold, corresponding to the large pores form PU template; (**e**) SEM images of the porous structure of a strut; (**f**) cross-section of a strut.

All of the HHA scaffolds fabricated were highly porous, with porosities of approximately 95% as calculated using Equation (1). As shown in Figure 1a,b, the overall porous structure closely resembled the architecture of the PU foam template. The interconnected pores of the HHA scaffolds were primarily in the range of 400–600 µm (Figure 1c,d), and the struts of the scaffolds were about 35–60 µm across (Figure 1e). The surface morphology and the hollow nature of a HHA strut are shown in Figure 1e,f. It was estimated that the thickness of the strut wall was about 2–10 µm. The void in the center of the struts results from removal of the PU template and has been observed in other sintered ceramic foams obtained by using the replication method [6].

The pore size for bone tissue engineering scaffolds depends on the type of material; however, it is generally accepted that the pore size suitable for mineralized bone ingrowth is in the range of 400–600 µm [25]. Large pores are also believed to favor vascularization. When porous hydroxyapatite scaffolds were implanted into rats, alkaline phosphate activity, osteocalcin content and bone ingrowth were more apparent in 300–400 µm pores, and this was the critical size above which blood capillaries were observed [26]. In addition to pore size, Otsuki et al. suggested that the interconnectivity of pores inside the porous scaffolds should be considered as another important factor [4]. Hence, besides mandatory biocompatibility and biodegradability, the scaffolds should have a suitable pore size, and a highly interconnected porous structure. In essence, the windows between pores must also be of critical size. The HHA scaffolds fabricated in this study showed a distribution of interconnected, open pores with diameters of 400–600 µm (Figure 1b,d), which are likely to be beneficial in facilitating cell infiltration, bone ingrowth and vascularization. These interconnected large pores are a direct result of employing a PU foam as a template.

### 3.2. Nanoscale Porous Structure

**Figure 2:**
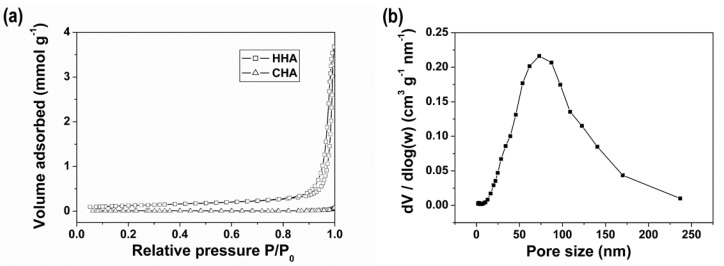
(**a**) Nitrogen sorption isotherms of HHA and CHA scaffolds; (**b**) the BJH-adsorption pore size distribution of the HHA scaffold.

Figure 2 shows the nitrogen sorption isotherms of HHA and CHA, and the pore size distribution of HHA calculated from the adsorption branch using the BJH model. The BET surface areas were 9.6 m^2^ g^−1^ for HHA and 0.6 m^2^ g^−1^ for CHA. The HHA scaffold presented a broad pore size distribution, with a pore diameter of around 75 nm at maximum pore volume. It has been previously demonstrated that pores in the nanometer range are beneficial for osteogenesis, because this characteristic favors cell adhesion and the adsorption of biologic metabolites [27,28].

**Figure 3:**
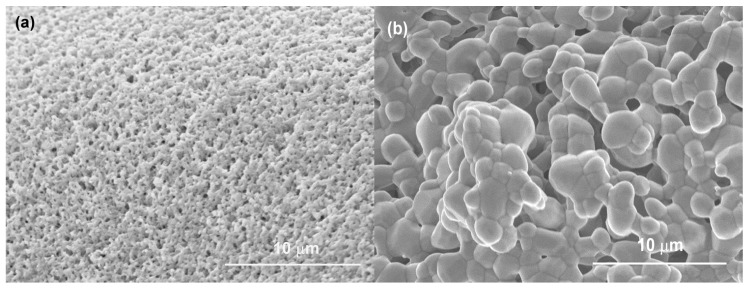
SEM images showing surface morphologies of (**a**) HHA calcined at 720 °C for 5 h; (**b**) CHA calcined at 1100 °C for 3 h.

As shown in Figure 3a, the surface of HHA was highly porous and composed of many nanoscale pores. However, the surface of CHA was quite dense and large hydroxyapatite crystals of 2–5 μm were observed (Figure 3b). During the synthesis of CHA scaffolds, the loosely contacted HA powder required high-temperature sintering to produce a mechanically stable structure. As a result, substantial crystal growth occurred during this process giving the denser structure and lower surface area. In contrast, the sol-gel synthesized HHA scaffold requires relatively lower processing temperatures, and therefore had a higher surface area and smaller crystals, despite its longer calcination time.

### 3.3. Crystallization and Mechanical Properties

**Figure 4:**
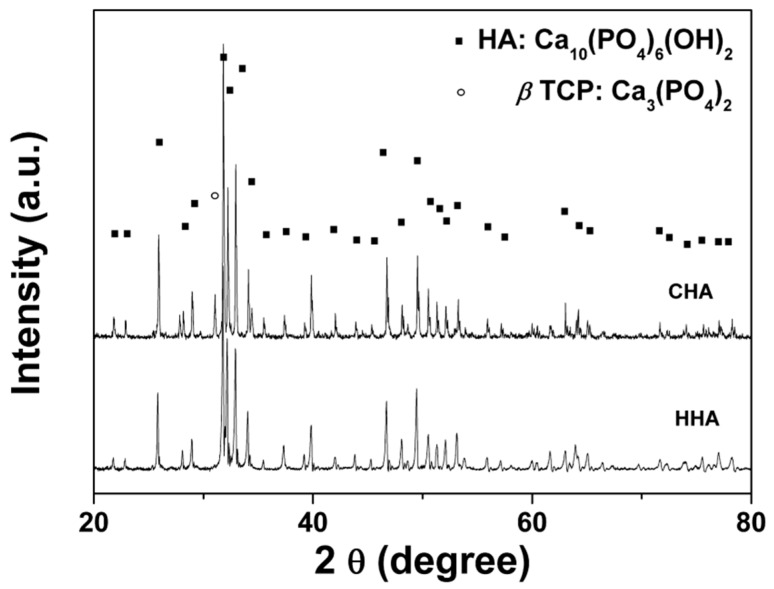
XRD patterns of the HHA scaffold calcined at 720 °C and CHA scaffold calcined at 1100 °C.

The XRD investigation revealed that crystallization had occurred for both scaffolds after calcination (Figure 4). Both the angular location and intensity of the peaks match the standard card (PDF 04-008-8714), which indicates that the major crystalline phase is HA. The XRD patterns further showed that there were no crystals of calcium hydroxide (Ca(OH)_2_), calcium oxide (CaO), tricalcium phosphate (Ca_3_(PO_4_)_2_), or octacalcium phosphate (Ca_8_H_2_(PO4)_6_·5H_2_O). Some impurities or amorphous phase may be present in the HHA, but at levels below the detection limit of the instrument used to collect the XRD patterns. For the CHA scaffold, there was a small amount of beta tricalcium phosphate (β-TCP) detected. At temperatures higher than 900 °C, partial decomposition of HA may take place resulting in β-TCP, which is commonly observed in sintered HA materials [29,30].

The compressive strengths of the as-synthesized scaffolds and the scaffolds after immersion in PBS for 1–14 days are given in Table 1. The difference in compressive strength for CHA and HHA before immersion in PBS was expected due to the increased level of porosity in the HHA scaffolds (95%) compared with the CHA scaffolds (89%). Nonetheless, the lower strength of 80 kPa was sufficient for the scaffold to be handled, such as manipulating the scaffolds during cell culture tests.

Theoretical modeling of the mechanical behavior of porous materials suggests that the compressive strength of a porous material depends on its porosity [31]. Higher strength may be obtained at the cost of reduced porosity. The strength of HHA scaffolds may be improved by applying more layers of coating. Only three HA layers were applied to the template in this research.

The in vitro degradation process of HHA was tested by immersing the scaffold in PBS for up to 14 days. The compressive strength of the HHA scaffold decreased over 1–3 days immersion, after which there was little variation. The decrease of compressive strength of the HHA scaffold in PBS might be related to the presence of small amounts of impurity (such as CaO, Ca_3_(PO_4_)_2_) or amorphous phase in the HHA. Dissolution of the impurity or amorphous calcium phosphate may have compromised the HHA scaffold integrity, and so caused a decrease in compressive strength. It should be pointed out that scaffold degradation data obtained from in vitro experiments might be significantly different to that from in vivo. For instance, Tamai et al. reported that the compressive strength of a HA scaffold significantly increases (e.g., from 10 to 30 MPa) due to tissue ingrowth in vivo [32]. Commercial porous HA blocks (Kunshan Chinese Technology, porosity 85%) exhibited a compressive strength of 2.5 MPa. It is apparent that the strut of the HHA scaffold is hollow, i.e. there is the void left behind by the porous polymeric template after its burning-off. This could be one of the reasons why the mechanical strength of these scaffolds is so low. However, our HHA scaffolds are designed to allow enhanced cell proliferation (discussed later) and cellular infiltration, as well as osteoconduction and vascularisation, so that the mechanical integrity of the scaffold improves with time.

### 3.4. Protein Adsorption

The initial event occurring upon implantation of biomaterials is the adsorption of a monolayer of proteins from the surrounding body fluids. The adsorbed proteins on the surface influence the precipitation, crystallization, and growth of calcium phosphates, as well as in vivo mineralization [33,34,35]. Moreover, the proteins affect the subsequent cellular response to the material, and thus the overall performance of the implant [34].

**Figure 5:**
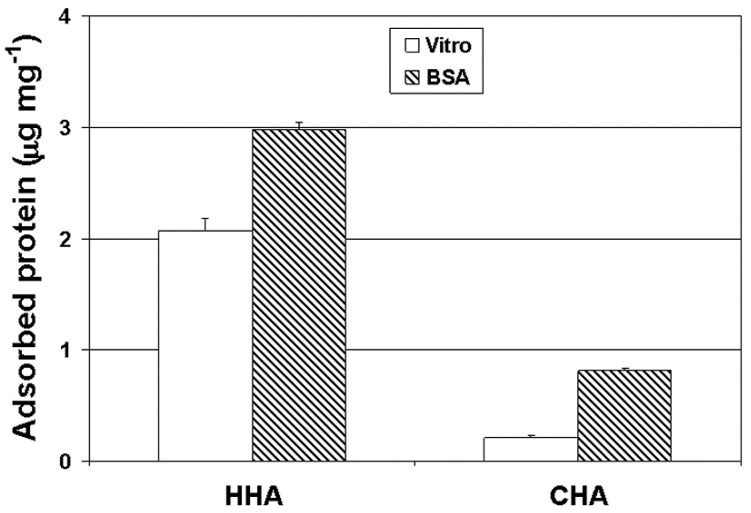
BSA and vitronectin (Vitro) adsorption on HHA and CHA scaffolds after incubation in 0.2 mg mL^−1^ of the respective protein at room temperature for 4 h.

As presented in Figure 5, the HHA scaffold showed significantly higher adsorption of both BSA and vitronectin over CHA (3.8-fold and 10-fold, respectively). The greater protein adsorption was attributed to the higher surface area of the HHA scaffold (16-fold higher than CHA) and the presence of nano-sized pores on the surface (Figure 1e); the pores were considered to make more HA surface accessible to protein adsorption. Vitronectin, a serum protein, is known to mediate adhesion of osteoblasts on substrate surfaces [36,37]. The finding that the HHA scaffold adsorbed a greater amount of vitronectin than the CHA scaffold is consistent with the subsequent enhanced adhesion and proliferation of osteoblasts on these scaffolds (as discussed below).

### 3.5. Cell Proliferation and Biocompatibility

**Figure 6:**
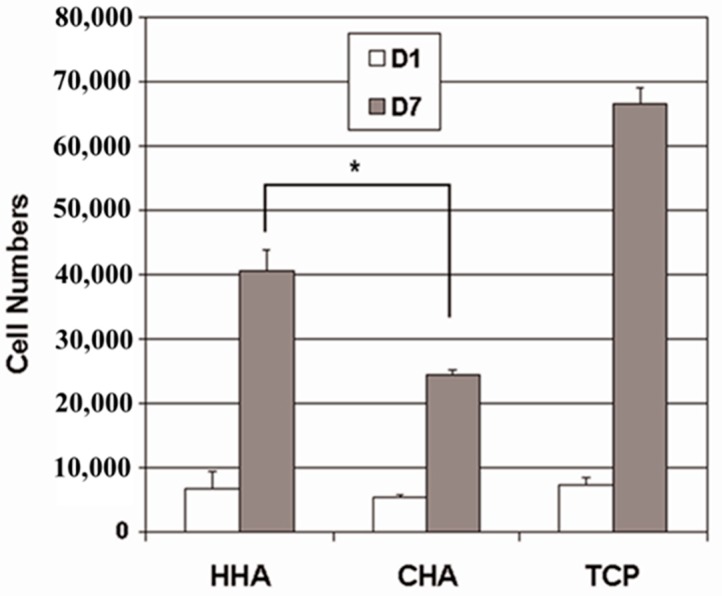
Proliferation of Saos-2 osteoblast-like cells on HHA and CHA scaffolds, and tissue culture plate (TCP) after 1 and 7 days (D1 and D7, respectively). * Significant difference, *p* < 0.01.

Saos-2 osteoblast-like cells bound comparably to HHA, CHA and control tissue culture plates (TCPS) after 1 day of culturing (Figure 6). However, after 7 days in culture, the number of Saos-2 cells on the HHA scaffolds increased 6 times over that initially seeded (day 1), while the Saos-2 cells proliferated slower on the CHA scaffolds, with the cell number increasing only 3.7 times over the same period. That the proliferation of Saos-2 cells was significantly greater on the HHA scaffolds than on the CHA scaffolds implied that the HHA scaffolds provided a better environment for the osteoblast-like cells to attach and grow. The MTS assay is a definitive quantitative measurement of the extent of cell proliferation. By definition, an increase in cell numbers for an anchorage-dependent line necessitates cell spreading and migration. The surface properties of biomaterials play a critical role in this biological response. The first event that occurs after implantation of a biomaterial is formation of a fibrin blood clot, which contains various proteins, around the surface. Later these proteins stimulate the recruitment of mesenchymal cells that differentiate into osteoblasts [37]. In addition, this preferential proliferation on HHA scaffolds is in keeping with the previous finding that HHA scaffolds adsorbed greater quantities of vitronectin, which is found in wound clots and associated with wound healing, and plays an important role in osteoblast attachment and proliferation.

**Figure 7:**
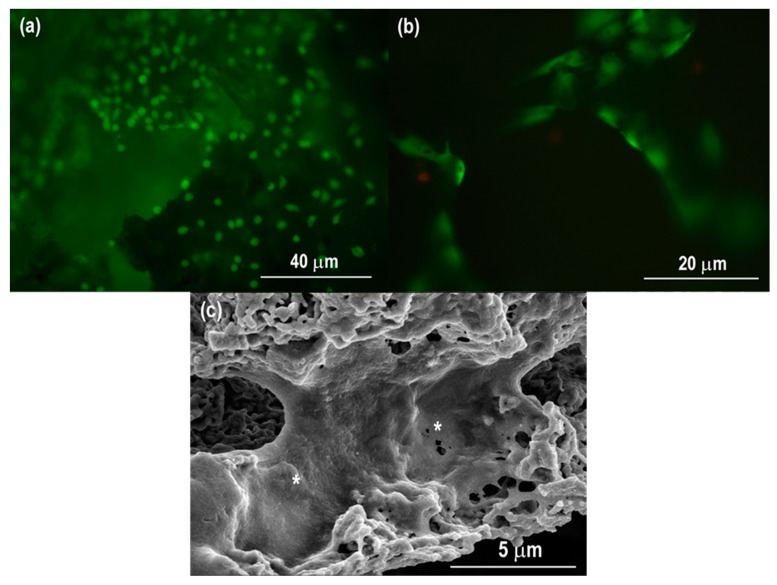
Fluorescence images from the Live/Dead^®^ assay (viable cells stain green; dead cells stain red) on (**a**) the edge and (**b**) in the center of the porous HHA scaffold disc. (**c**) SEM image of Saos-2 osteoblast-like cells on the HHA scaffold after culturing for 7 days. * Cells apparent on the wall of the HHA scaffold.

Fluorescence and SEM micrographs of Saos-2 osteoblast-like cells on the porous HHA scaffolds after cell culture for 7 days are shown in Figure 7a,b. The Saos-2 cells attached, and can be seen to have spread well on the surfaces of the HHA scaffold. The Saos-2 cells also grew along the strut of the porous HHA (Figure 7b). Figure 7c shows the cell morphologies under SEM to provide a closer observation of the cell adhesion and spreading. The results of the cell morphology and attachment observations accord with both the results of the MTS cell proliferation assay and the protein adsorption assay. The above results strongly indicate that HHA scaffolds exhibit well in vitro biocompatibility and promote cell proliferation.

## 4. Conclusions

Hierarchically porous hydroxyapatite scaffolds (HHA) were synthesized through sol-gel chemistry and a templating technique. In comparison with conventional hydroxyapatite scaffolds produced through powder sintering techniques, the sol-gel method offers the use of lower sintering temperatures, chemical purity and larger surface areas. Templating with a polyurethane foam included large open porosity in the HHA scaffold, with pore diameters of 400–600 µm. By incorporating a tri-block copolymer, Pluronic F127, nanometer pores were introduced into the scaffolds, which contributed to greater surface area and an enhanced capacity for protein adsorption. Furthermore, osteoblast-like cells attached and proliferated well on the HHA scaffold, signifying the biocompatibility of the scaffold. Thus, a template-assisted, sol-gel route is a feasible alternative approach for designing and synthesizing novel scaffolds for bone tissue engineering and other potential biomedical applications.

## Figures and Tables

**Table 1:** Compressive strengths of the CHA and HHA scaffolds as-synthesized, and for HHA after degradation in PBS for 1–14 days.

Characters	CHA	HHA	HHA—1 day in PBS	HHA—3 days in PBS	HHA—7 days in PBS	HHA—14 days in PBS
Porosity (%)	89	95	97	98	98	98
Compressive strength (kPa)	210 ± 20	81 ± 10	32 ± 8	26 ± 8	28 ± 9	26 ± 8
